# Section on Diseases of Children

**Published:** 1881-06

**Authors:** 


FIRST DAY.

DISEASES OF CHILDREN.—Chairman, Dr. A. Jacobi, of New York ; Secretary, Dr. T. M. Rotch, of Boston, Mass.

The following is an abstract of the paper of Dr. H. I. Bowditch, of Boston, entitled "*The Relation between Growth and Disease.*"

After discussing the various influences which must be taken into consideration in approaching this important subject, such as climate, race, social condition, occupation, and sex, Dr. Bowditch directed the attention of the medical profession to carrying out more extended observations on this subject, especially in the West, where the emigrant class, coming from different countries, present especial advantages for this kind of investigation. He also spoke of the intention of the Massachusetts State Board of Health, Lunacy and Charity, to distribute blank cards and circulars of instruction to aid in this work, and it is probable that the National Board of Health will soon undertake a similar work on a larger scale. Dr. Bowditch said that it seemed probable that the accurate determination of the normal rate of growth in children will not only throw light upon the nature of the diseases to which childhood is subject, but will also guide us in the application of therapeutic measures. The statistics of growth, taken in connection with those of disease, might very possibly reveal unexpected relations between periods of slow and rapid growth, and the ages at which certain diseases most frequently occur.

He referred to Dr. Percy Boulton's expression of "danger signal" in his paper on the weighing of children. Dr. Bowditch then exhibited a chart representing the case of a child, between two and three years old, where careful and systematic weekly weighing showed, first, the approach, by some weeks, of a chronic disturbance of nutrition, represented by enlarged cervical glands and clay-colored stools; and second, after recovery, the approach of an attack of measles, the "danger signal" of progressive loss of weight preceding the eruption by at least a week.

Dr. Billings spoke of the value of Dr. Bowditch's paper, and presented to the Section the statistical cards intended for circulation by the National Board of Health.

Dr. Lee, of Baltimore, remarked that he had paid especial attention to this subject, and that he had noticed that a female child could lose more in proportion to its weight, without detriment to its health, than the male child of the same age. He also said that if the loss of weight preceding the eruptive disease was excessive, the case was so much more grave in its prognosis, and that this loss of weight preceded this eruption by from four to five days.

Dr. Busey, of Washington, read a paper entitled "The Relation of Meteorological Conditions to the Diarrhœal Diseases of Children."

Dr. White, of Boston, presented a paper entitled "Some of the Causes of Infantile Eczema, and the Importance of Mechanical Restraint in its Treatment."

He first described the many and varied external influences which immediately affect the delicate skin of the new-born, as being a common cause of eczema, and laid especial stress on the fact that heat was the more usual cause of the disease than cold. He said, however, that these external influences furnish but a small proportion of all the cases of the disease which occurred at this period of life, although by far the greater part of those concerning the etiology of which we have any positive knowledge. During the last twelve years he had treated at the Massachusetts General Hospital 5,000 cases of eczema, of which 1,770 occurred in children of ten years of age and under, and of which the largest proportion, viz: 569 cases, was in the first year of life. He said, eliminating the operation of the causes directly acting upon the skin from without, above mentioned, and a few other extraneous agencies, the parasitic chiefly, that he did not hesitate to say that he knew nothing whatever of the causes of the disease in the remainder; also, that as far as his experience went, eczema affected all classes of society alike, occurred at all seasons of the year, came in children of all degrees of health, in the perfectly sound as frequently as in the feeble; that it had no necessary connection with any other disease of childhood; that it showed itself in an equal proportion in bottle babies and those reared at the breast, and was independent of diet; also, that if there were other assigned causes, he would here say that his observation gave him no justification for believing any of them.

He stated that there was no more necessity for a supposed sympathy with or dependence upon the state of the blood, or the condition of some other organ, when the skin is affected with eczema, than when the lung, kidney, etc., is affected. He then, after speaking of the extreme suffering which the little patients undergo, said that the prime factor of the treatment was the prevention of scratching, and he described what he considered to be the proper method of controlling the child's movements, viz: a system of swathing in a pillow-case, by which the same chances of success in the therapeutics of infantile eczema is given, as exist in the adult.

He finally said that when the strait-jacket treatment is carried out, the child soon becomes used to the confinement, and a wonderful improvement takes place, not only in the disease itself, but in the morale of the family, which always becomes painfully disorganized during the existence of the disease; and he mentioned, as an important factor in the success of the treatment, that a grandmother (if present), in every case of infantile eczema, is the first evil to be eliminated. In conclusion, he considered that the simple mechanical means found in every household included all that was important in the treatment of one of the most distressing and rebellious diseases of infancy.

Dr. L. Duncan Bulkley regretted criticising the paper, as he felt obliged to do, in the absence of his friend Dr. White, but the views presented differed so radically from those which he had formed from experience that he could not help so doing, because he felt that the subject demanded it. For those who did not know of his acquaintance with the disease, he would state that he had recently made analyses of 2,500 personal cases of eczema, and of these nearly 700 had occurred at an age which classed them as infantile eczema: he therefore believed that he could speak with authority on the subject. Dr. White had, it is true, treated of only one feature in the management of the disease, namely, physical restraint, but he believed criticism to be called for because of the principles which underlie, or which call for this element of treatment; the premises being wrong, what follows must of necessity be wrong.

The speaker believed that Dr. White here, as elsewhere, laid far too great stress upon local causes of eczema, and ignored entirely the influence of internal, general, dietary, and hygienic causes; if these are not recognized and managed, the results of local treatment are imperfect and uncertain. He did not believe that the children with infantile eczema were really in perfect health, but that always the evidences of imperfect assimilation, could be discovered: the evacuations from the bowels were faulty, the urine constantly presented evidences of mal-assimilation, and searching investigation would always demonstrate imperfect health in the child. In nursing children, the mother should always receive very careful attention, as very commonly she would exhibit dyspepsia, or constipation, or perhaps is taking ale, beer, much tea, etc., which disagree and cause trouble in the child; or perhaps she was very much debilitated, etc.

This subject could not be fully entered into here; the speaker expected to read a paper, on "Diet and Hygiene of Eczema," before another Section at the present meeting. He was, however, absolutely convinced of the importance of this matter, and that if attention was thoroughly paid to it much less would be required locally, and what was used would be more rapidly and completely successful.

Internal treatment to a certain degree was also absolutely necessary. And without it, physical restraint, as any local treatment, would be comparatively ineffective. This could not be discussed here, but he would only mention in illustration the internal use of small purgative doses of calomel, every other day, and a mild alkali, as acetate of potassa in the liquor ammoniæ acetatis, with a little nitre and perhaps aconite. Individual cases required very different management.

In regard to the method of restraint proposed in the paper, he would say that he had never employed such restraint, simply because he had never found it necessary. If the itching is relieved, the restraint is not required, because the habit of scratching is soon overcome when the infant finds that a proper application gives relief. If the itching is not relieved, such confinement is torture beyond any description, judging from the statement of older patients, who cannot abstain from it by any force of will, and who assert that they would scratch even if they died from it. The agony of little ones mechanically restrained is fearful to see.

In the paper the writer had mentioned the use of diachylon ointment. This the speaker very rarely employed in infantile eczema, as he believed it very inefficient in arresting itching. Tar in some form was far more efficacious; indeed, the speaker said that he had little to desire in the way of an application to infantile eczema beyond the following ointment. Recipe: Unguenti picis, one ounce; zinc oxid., two drachms; unguenti aquæ rosæ, three ounces. Mix. This should be very carefully prepared and very thoroughly and abundantly applied. If it appears stimulating, less of the tar ointment may be used. He laid great stress upon employing the rose ointment and not simple cerate, or lard, or vaseline or petroleum. The ointment should be made of a consistency to spread easily and yet not to all melt away after application.

The writer had spoken of removing the restraining bands in order to wash the surface. The speaker was very positive in the directions given in regard to the use of water to eczematous surfaces in children: they were only to be washed according to direction, and that very rarely, often only at intervals of several days; moreover, it was all-important that the protective ointment should be replaced *immediately* after the surface is dried, and renewed sufficiently often to keep the parts completely protected, even twenty or more times the first day. On covered parts the ointment may be thickly spread on the woolly side of sheet lint and bound on. Among the hundreds of cases the speaker had never covered the face with a mask, and had rarely been obliged to restrain the infant much after the first day or so. The only restraint he had ever practiced was putting on muslin mittens, tied around the waist, and then tapes from these passed behind the back or beneath one leg. He had never employed a mask.

Under the management thus briefly indicated, if every point could be carried out, there was but one result, arrest of the eruption, and if dietary and hygienic elements were persisted in, a cure of the disease. The attention to the mothers of nursing children he considered most important.

Dr. Ulrich, of Pennsylvania, in discussing the paper, said that he fully agreed with Dr. Bulkley, and that he entirely opposed Dr. White's treatment. In a long professional career he had never used a system of restraint in these cases, and that he would prefer to knock the little patient on the head at once rather than submit it to the torture's of Dr. White's strait-jacket.

Dr. H. O. Marcy exhibited to the Section some elastic double canular injection tubes which he thought useful in various diseases. The tubes presented were of different sizes to be used in inflammatory conditions of the uterus, bladder, rectum and bowels. They are not costly, easy to carry and very serviceable. Dr. Gouley, of New York, asked Dr. Marcy if he claimed this a new device; Dr. Marcy replied that they were new to him. Dr. Gouley had used certainly the catheter years ago, and protested against the introduction of so many so-called new and valuable instruments; thought the next era would be time enough for more, and could not appreciate its great use in bladder troubles. Dr. Marcy thought there must be virtue in hot water in diseases of the bladder as in diseases of the uterus. The stomach tube he feels much easier to introduce. Dr. Wm. A. Byrd, of Quincy, Ill., thought the stomach tube very valuable. When called to see a child with convulsions from indigestion, introduces stomach tube, and with water washes out the stomach.

Dr. Wm. A. Byrd, of Quincy, Ill., presented a specimen of ulceration and perforation.

The following is an abstract of the paper by Dr. D. H. Goodwillie, of New York city, on Thumb-sucking, illustrated by the report of a case and exhibited by a wax model.

*Treatment* consisted in breaking up the habit by applying a leather pad to the elbow, preventing the hand from coming to the mouth.

*Nasal Catarrh* by douches and the application of powder blown into the nose, proper food, clothing and rest.

His conclusions were as follows:

(1.) Thumb-sucking is more disastrous to the health of the child than the sucking of the other fingers, for the thumb, once in the mouth, it more readily remains during sleep.

(2.) It interferes with the child's proper rest, which should be continuous and undisturbed, and so becomes a source of nervous irritation and exhaustion.

(3.) It interferes with the natural respiration through the nose, and sets up abnormal conditions.

(4.) It malforms the anterior part of the mouth, and effects proper mastication.

At the conclusion of the reading of this paper, the Section adjourned until 3 o'clock, P. M.

SECOND DAY.

DISEASES OF CHILDREN.—Chairman, Dr. A. Jacobi, of New York; Secretary, Dr. T. M. Rotch, of Boston, Mass.

Dr. Jacobi spoke of the discussion on *Eczema* which had occurred at the last meeting, and remarked that he supported Dr. White, of Boston, in controlling scratching by mechanical measures. He said that if scratching was prevented, children would recover as easily as adults, and that although the procedure was somewhat severe, still the mother and nurse would be saved a great deal of trouble. The mask for the head had proved very serviceable in his hands. As regards Dr. Bulkley's views, he thought that the constitutional causes were nothing more than we have in any affection. Dr. Jacobi agrees with Hebra that no water should be used. Very rarely, and in special cases, the judicious use of water may be of service. The main point is to remove the scabs. For this purpose potassa soap, very soft, may be used; or liquor of caustic potassa, with ten or twelve parts of olive or castor oil, can be applied twice, three, four, or five times during the day, and will produce remedial effects very quickly. The surface left exudes the serum, which hardens and forms the scab. This serum must be touched and taken up with a soft towel, and finally astringents are applied to the raw surface. These applications can be made to please the fancy of the physician. Zinc and diachylon plaster are both valuable. A number of cases will not be cured, and then we must resort to constitutional treatment. The milk of the nurse must be rendered digestible, and it will be found that many children will thrive better on artificial food than on the milk of the mother or nurse. A good general condition of the system must be obtained. To say that an eczema can be cured forever, is perhaps going too far, for we see cases repeatedly coming under our observation as cured. Just as in insane asylums we notice cases that are published several times as "cured," so it may be in eczema or any other intractable affection.

Dr. Jacobi, in the absence of Dr. R. J. Nunn, of Savannah, read a paper for him on the *Treatment of Diphtheria*. Dr. Nunn approached the subject with timidity. The disease had raged in Savannah with very fatal effects, and a letter from a friend elsewhere said that, after treating 600 or 700 cases, his faith in the efficacy of drugs was very feeble. The causes will probably remain speculative for a long time. Is the disease the same always and everywhere? The causative influences are probably not the same in all cases. Medicines which cure the disease in Germany fail in this country, and the discussions as to the identity of croup, diphtheria and scarlet fever are strong arguments in favor of this belief, and all treatment based upon one cause must fail to relieve all cases. Dr. Nunn quoted Dr. Jacobi as saying: "The entrance of the diphtheritic poison into the system is not the same in all cases." . . "There are cases in which the origin of the disease is decidedly local." . . "There are others in which the poisoning of the blood through inhalation is the first step in the development of the disease." A powder used by Dr. J. B. Read is as follows: Sulphur sub., grs. xlviij; acid tannic, grs. xij; acid salicylic, grs. i; pulv. potass. chlor., grs. xij. Precaution must be used in compounding this prescription. A little of this powder is put on the back of the tongue every hour or two, and a small piece of ice given afterward. It will be seen that this prescription is a combination of antiseptics principally.

In another case treated by Dr. Nunn, the following formula was used with good effect: Sulphur, grs. vij; acid boric, grs. iv; acid tannic, grs. j; acid salicylic, grs. j; resorcin, grs. j. Another formula is: Sulphur sub., grs. viij; acid boric, grs. iv; acid benzoic, grs. j; acid salicylic, grs. j; acid tannic, grs. j; acid tartaric, grs. iv; sodii chlorid, grs. iv; resorcin, grs. j.

Dr. Nunn thinks that this formula will prove efficacious.

Dr. Lathrop, of New Hampshire, has experimented with chloroform largely, and finds it a highly useful agent. He uses it in diphtheria and other throat affections on a piece of cotton attached to a tube or pen-holder. The cases usually required visiting no longer than four days, but the cases were not so malignant as had been reported in other localities.

Dr. Lathrop stated that no unpleasant effects have ever followed this plan of treatment, and that the child, in true diphtheria, would not complain of *smarting* from the application of chloroform.

To a question from Dr. Jacobi, Dr. Lathrop said that he had used this plan of treatment in one hundred cases. Of course, constitutional measures are added.

Dr. William Lee, of Baltimore, has used equal parts of tinct. ferri chlorid. and ol. ricini with benefit. He considers the disease as local at first, and then constitutional. A physician from his county had used large doses of ol. copaibæ with benefit. An emetic was then given to remove the membrane

Dr. G. Vivian, of Minnesota, has used, in severe epidemics, alcohol as an inhalation, and has employed as much as a quart of alcohol a day. He has never seen any constitutional effects ensue.

Dr. J. McNeal, of Gettysburg, Pa., recommends the following: Potass. bromid., ʒi, potass. chlorat., ʒiij, acid carbolic, grs. xx, aq. ℈i. Use in an inhaler. Locally, chloroform ʒij. lin, saponis ℥i—ʒij.

Dr. F. E. Hitchcock, of Rockland, Maine, uses equal parts of sulphurous acid and water in an atomizer. The proportions can be varied, and the acid used as a gargle. Cold affusion externally.

Dr. Jacobi, in answer to an inquiry concerning the benefit of pilocarpin, said that his opinion of it was unfavorable. It was proposed as a specific by a Dr. Guttman; and while on this subject he would state that this gentleman was not Dr. Guttman, of Berlin. The article on pilocarpin had struck him as nothing more than an advertising arrangement. In diphtheria there are two forms—one in which the deposit can be readily separated from the mucous surface beneath, and one in which the deposit is deeply embedded in the lower structures. In the latter form Dr. Jacobi believes that pilocarpin does harm, and in one case he thinks that death was hastened by using this drug. Pilocarpin debilitates the heart's action by giving rise to nausea and vomiting. The milder cases of diphtheria recover, as a rule, if left alone, and in all cases of the disease he thinks the utility of pilocarpin doubtful. The paper in which pilocarpin was recommended as a specific is not satisfactory as regards the description of the cases treated, and to say that this and that remedy is a specific is certainly not dignified. If the remedy be used at all, a fluid extract is the best preparation, as the muriate of pilocarpin is decomposed in the stomach.

Dr. Jacobi, also, in answer to a question from Dr. Lee, concerning the effect of lime, said that his opinion of this agent was not so high as that of many writers, but that he would speak more fully of both lime and pilocarpin in his article on this subject.

Dr. E. H. Bradford presented a paper entitled "Re-section of the Tarsus in Severe Cases of Club-Foot," of which the following is a summary:

Dr. Bradford first reviewed the literature of the subject, and then reported two cases on which he had operated.

The first case was a girl, 11 years old, with severe equinovarus, the axes of the foot being at right angles with that of the leg.

Tenotomy and mechanical treatment were tried for a month with but slight benefit. Dr. Bradford removed a wedge-shaped section of bone from the tarsus with a metacarpal saw, and, with antiseptic precautions, there were no constitutional symptoms after the operation, excepting that the temperature rose once to 101°, being otherwise normal during the recovery. The wound healed under the blood-clot, as occurs with a thorough antiseptic dressing, and in five weeks the patient was able to walk without a cane.

She remained in the hospital for some time under observation, and was discharged with the foot nearly in a normal position.

The second case was a boy, 13 years old, with double congenital club-foot of aggravated type. This first foot was operated on November 9, and was sufficiently well to bear his weight by December 12, and was entirely healed by January 19. The second operation was performed January 9, and by February 4 he was allowed to walk on both feet. He remained under observation until April 2, when he could walk a mile without apparatus, and wearing ordinary shoes. The temperature rose to 102° on the second day of the first operation, but fell to the normal on the next day, and remained so. After the second operation, on the third day the temperature rose to 104°, but fell after removing the drainage tube, which was stopped, and there was no subsequent rise. The plaster of Paris bandage was made use of after the operation.

Dr. Bradford then described minutely the after treatment in this case, and stated that there remained, after recovery, about 30° of motion, and that the boy could stand on either foot, or raise himself on his toes.

In regard to the antiseptic precautions, Dr. Bradford thought that they offered the best safeguard, and that it must be admitted that a study of the reported cases justifies the impression that the danger is not so great as has been supposed.

The temperature charts, the photograph, a tracing of the left foot, and a cast of the right foot before and after operation, of the second case, were exhibited.

Dr. Jacobi remarked that Dr. Bradford's cases were exceedingly interesting and instructive, and that, as shown by the casts, they could not have been successfully treated without just such a procedure as Dr. Bradford had adopted.

The meeting was then adjourned, to meet again at the regular hour.

THIRD DAY.

DISEASES OF CHILDREN.—Dr. Blake presented a paper entitled "Middle-Ear Disease in Children in the Course of the Acute Exanthemata."

Dr. Blake first spoke of the frequency of the disease, as shown by the facts that 35.70 of all the cases of purulent inflammation of the middle ear occurring at the Massachusetts Charitable Eye and Ear Infirmary followed measles and scarlet fever, and of deaf mutes examined by the writer, 27.70 lost their hearing as the result of scarlet fever.

In both exanthemata the inflammation affects the mucous membrane, lining the middle ear; occurs during the acute stages of the primary disease, and runs its course quickly; hence its importance to those having to deal with diseases of children.

Dr. Blake then said that much might be done in the early stages to diminish the severity and shorten the duration of the inflammatory process, which, in children, owing to the greater vascularity of the mucous membrane, and the readier solution of continuity of the tissues, favoring ulceration, is usually more rapid than in adults.

In scarlet fever the aural complication may occur at any time; usually runs its course rapidly, and furnishes in a short time a well-marked acute purulent inflammation of the middle ear.

In measles there are two types, occurring early and corresponding to the acute catarrhal inflammations, following "head-colds," the other originating primarily in the membrana tympani, and accompanying the appearance of the facial eruption. The symptoms in that form occurring during scarlet fever, are—rise in temperature, pain at first occasionally, then constant, in very young children, shown by moaning, unrest, and a desire to press the affected ear against the pillow. These symptoms are of course due to increased pressure within the tympanic cavity from serous exudation, as is shown by the relief afforded by the use of the Politzer and douche, permitting the escape of a part of the fluid. The continuous pain later is often not similarly relieved, either because of serous effusion of the membrane itself, or more complete closure of the Eustachian tube.

Nature usually relieves the pressure by a spontaneous opening, but only after prolonged suffering and prostration on the part of the patient, or possibly serious injury to the transmitting structure of the middle ear. The opiate treatment is superficial. In the early stages, frequent gargling, and if pain is complained of, the air douche should be used frequently. Instillations of warm oils and poultices are objectionable, as they interfere with subsequent operative interference; if necessary, glycerine and warm water, two or three parts to one, and a dry cloth over the ear are better.

In a later stage, if pus is present, the evident remedy is puncture of the membrane at its most prominent portion with a lance, suture needle, or saddler's needle.

Dr. Blake then spoke of measles. In the first form, the remedies referred to in the early stages of scarlet fever are equally applicable here; if they fail, acupuncture and drainage are preferable to paracentesis, which may be done with a needle, and then a wick inserted, which serves the double purpose of withdrawing the serous fluid, and keeping the lining of the canal dry. Syringing should only be resorted to when the discharge becomes muco-purulent.

The second rare form is characterized primarily by a congestion of the membrana tympani, without congestion of the tympanic mucous membrane, and is not relieved by the use of the air douche. It is due probably to inhibition of the vasomotor nerves controlling the tympanic branch of the carotid artery.

These cases either resolve spontaneously, or the conditions described in the first type of inflammation of the middle ear becomes established. Instillation, dry warmth and acupuncture, may be resorted to with good effect. For general treatment the bromides are especially indicated.

Dr. Jacobi remarked that closing the mouths of infants and children, and simply blowing into the nose, is often a very valuable method of relieving severe earache, and that in a number of cases he had obtained most excellent results from this procedure, the cause of the trouble probably being a catarrhal affection of the Eustachian tube.

Dr. Jacobi remarked that his paper should have been read before the general session, but that on account of the crowding of business on the last day, he would read the paper to the section.

Dr. Jacobi said that the Children's Section was a new one, that he was anxious to see it a prosperous and interesting one, and that the sympathy and co-operation of the gentlemen were necessary to its success.

"The limited time at my disposal precludes my going over much ground. The remarks I shall make will, therefore, be confined to the progress made in our knowledge of the acute contagious constitutional diseases (rubeola, scarlatina, variola, and typhoid fever), and the acute contagious infections of the mucous membranes, such as dysentery and diphtheria."

The literature of the subject during the past year was described, embracing text-books, atlases and monographs, which were exhibited before the Section.

The concluding words of Dr. Jacobi on rubeola are as follows: "Evidently the latest contributions to our knowledge of rubeola give weight to its classification among the acute contagious constitutional affections." * * "The description of the symptoms varies a good deal with the authors. These differences are best explained by the fact that mild cases of either measles or scarlatina may not present all their usual symptoms, and by the other fact that during the prevalence of an epidemic of rubeola there is, as a rule, a contemporaneous epidemic of measles and scarlatina, and also diphtheria."

In such times cases of common nasal, pharyngeal, and laryngo-tracheal catarrh are very frequent indeed; thus there have been but very few infants and children in our large northern cities but were affected in this manner. Thus it happens that there are many symptoms belonging to the mucous membrane of the digestive and respiratory tract which invariably complicate the slightest other affections. Thus it is, also, that many authors have looked upon these accidental complications as if they were genuine symptoms. The very best authors—for instance, Gerhardt—when they mean to describe roseola, depict mild or sometimes even severe cases of measles. Nasal catarrh, conjunctivitis, facial œdema, glandular swellings, belong to this class of symptoms, which, while they often occur in rubeola, do not belong to it by any means. The disease is very contagious. Its incubation lasts from two to three weeks, and is not attended with any fever. Now and then there are, during a period of from twelve hours to two or three days, prodromata, a mild increase of temperature, sometimes a catarrh of the mouth. The tongue is apt to show a few red marks along the margin, mainly in its anterior portion. The eruption, according to what I have seen, does not—contrary to Gerhardt's observations—begin in the face, but on the trunk, or more frequently on the extremities, mostly on the thighs and knees. Head and feet are not often affected. It looks very much like that of measles, sometimes not rising above the level of the skin, sometimes, however, rising above it in the manner of an urticaria. It seldom lasts longer than three days, but relapses on the fourth or fifth day are frequent. Desquamation begins early, now and then on the third or fourth day. Relapses may take place while desquamation is undisturbed in some localities. The temperature seldom exceeds 102° or 103°; it becomes normal about the fourth day. The prognosis is absolutely good, and the treatment either none at all, or expectant. The diagnosis, however, may be very difficult, and even impossible in an individual case, inasmuch as scarlatina, and particularly measles, are apt to be rife at the very periods of the rubeola epidemic, and their symptoms are complications too mild to be estimated at their full value. This fact, however, does not militate against the independent nature of rubeola, for it has never claimed that either measles or scarlatina can and must be recognized in every instance."

Speaking of *diphtheria*, Dr. Jacobi said: "In this connection I desire to say a final word in regard to large doses of chlorate of potassium, often recommended in diphtheria. My warnings in regard to this drug have at last been heeded. Extracts from my writings on this subject have been extensively published, and experiments on animals made in Europe by Marchaud and others have proved my clinical observation of the frequent occurrence of nephritis, and fatal nephritis, resulting from the incautious use of the potassium chlorate. A number of fatal cases have been described, and it may be that much carelessness on the part of the public and many accidents will be avoided in future."

In the article on *typhoid fever* the same writer says: "With reference to the effect of the bathing in typhoid fever he appears to have made the same observations detailed by myself some years ago in a lecture on typhoid fever published in the *Medical Record* of 1879. Whenever circulation is deficient, particularly when the patients have been anæmic at the onset of the disease; whenever, after the cool bathing, the feet do not get warm as quickly as the rest of the body, the end for which the bath was given is not attained. In order to reduce the temperature of the body permanently it is necessary that the circulation of the skin be restored very soon, and uniformly. If such be not the case no radiation from the external surface of the body can take place, and an undue amount of heat is retained within the body. It may then occur that the surface is quite cool while the temperature in the rectum is very high. In such cases, in order to reduce the temperature, I have had to plunge the patient into hot water for the purpose of restoring the cutaneous circulation. When, in such patients, cold water is to be used, the only proper mode of applying it is by packing or sponging. In these cases care must be taken that only the trunk, beside the head, is made the subject of local treatment."

In the recurrent cases to which he referred, Dr. Rotch said that reliance was not placed on the eruption alone, but on the general course of the disease, namely, a regular prodromal stage with coryza, conjunctivitis, and bronchial catarrh, followed by a characteristic eruption and a stage of desquamation, and that as these cases had been met with and reported by competent observers, there was no more reason why they should not be called a second attack of measles than when the same symptoms occurred in a patient for the first time.

Dr. Atkinson, of Baltimore, said he had undoubtedly seen numerous cases of recurrent measles.

Dr. Stiles, of New York, had seen cases where true measles had attacked the same children twice.

Dr. McDonald considered that the cases spoken of by Dr. Stiles were merely relapses of the original disease.

Dr. Jacobi agreed with Dr. Rotch that the term rubeola should be used for this disease, which simulates measles, roseola being different, as in typhoid fever.

He then said that he had frequently seen cases where what he considered the symptoms of true measles had occurred, and an exact recurrence of these symptoms years afterwards, and that he called the second attack true measles, and considered that measles could recur two, three or four times ; and he would beg leave to repeat the old story : " It eats like a toad, it looks like a toad, it creeps like a toad, hence why not call it a toad."

Dr. Adams, in referring to Dr. Jacobi's remarks regarding the great contagiousness of measles, described the admirable method of isolation in these cases in the Children's Hospital in Washington.

Dr. McDonald, of West Virginia, said that he had recently treated a large number of cases which differed in some respects from rubeola. He called it roseola, and the name became very popular with those who had the trouble.

Dr. Leigh, of Petersburg, reported two fatal cases of poisoning from chlorate of potash. The first was the case of a child three years of age with diphtheria. The mother gave the child one-half ounce of chlorate of potash, and the child died within twenty-four hours.

The second case was a child five years of age with croup. It took six drachms of chlorate of potash in twenty-four hours ; cyanosis appeared in six hours ; hæmaturia and death in forty-eight hours.

Dr. Potter remarked that in quite a large number of cases in his practice he had met rubeola and diphtheria occurring together.

Dr. Jacobi said that he had no doubt but that a number of deaths had occurred from chlorate of potash. When the blood is examined the corpuscles are changed, broken up and irregular, and there is a conglomeration of hæmatin scattered about. There is no positive poisonous dose : 3½ drachms given for five or six days killed a robust boy of fourteen years. Dr. Jacobi had taken himself half an ounce, and this dose was followed frequently by micturition.

Dr. Fontaine knew a patient who took one ounce and died. Chlorate of potash is soluble in 18 or 20 parts of water. Dr. F. does not use more than a grain to a child under one year, or 1½ to an adult, in twenty-four hours.

In answer to a question as to whether measles ever occurred twice in the same person, Dr. Rotch stated that during the epidemic which had lately been prevailing in Boston the disease (measles) had been observed to occur not only twice, but three times in the same person.

Dr. Shelden, of Norfolk, in a long professional career had never seen measles recur.

Dr. Lee, of Baltimore, expressed surprise at Dr. Rotch's statement, and said that in his opinion true measles never recurred, and that where a second attack was apparently present, close investigation showed that it was the diagnosis which was at fault, and that the eruption, although simulating that of measles, was really that of the roseola spoken of by Dr. Jacobi in his paper. He also inquired of Dr. Rotch whether he could distinguish the eruption of measles from that of roseola, and from the eruption which apparently followed certain articles of diet, as crabs ?

Dr. Rotch answered that he would first say, in order that there should be no confusion of terms, that there was a true measles and a rubeola (the rötheln of the German writers), as stated by Dr. Jacobi, and that so far as the eruption was concerned, it was often impossible to make a distinction ; second, that roseola, which was usually a papular eczema, had nothing to do with the other diseases, and that in the great majority of cases the eruption caused by eating crabs was that of urticaria, which of course was easily distinguished from the eruption of measles ; third, that he would again state that undoubted cases of rötheln had been observed in Boston, where the disease is well recognized and differentiated from measles.

---

[The following should constitute the closing part of the third day's proceedings in General Session.]

NOMINATIONS.

The following report was read by Dr. Stone, of Minnesota:

The Committee on Nominations met at Wilkinson Hall, and organized by electing Dr. A. Coles, of New Jersey, as Chairman, and Dr. Stone, of Minnesota, as Secretary.

The following nominations were made:

*For President*, Dr. J. J. Woodard, U. S. A.

*For Vice-Presidents*, Doctors P. O. Hooper, Arkansas; L. Conner, Michigan; Eugene Grissom, North Carolina; and Hunter McGuire, Virginia.

*Secretary*, Dr. Wm. B. Atkinson, Pennsylvania.

*Treasurer*, Dr. R. J. Dunglison, Pennsylvania.

*Librarian*, Dr. Wm. Lee, Washington.

St. Paul, Minnesota, was selected as the place for the next Annual Meeting; and Dr. Stone was appointed Chairman of the Local Committee of Arrangements.

Dr. Billings, U. S. A., moved the adoption of the report of the committee; paid a high compliment to Dr. Woodward, the nominee for President, and thanked the committee, in behalf of the Medical Staff of the Army, for the high compliment paid it in the selection of one of its most honored members for the highest position in the gift of the Association.

The report of the committee was adopted unanimously.

A letter was received from H. C. Parsons, Vice-President of the Richmond and Alleghany Railroad, inviting the members of the Association to take a short trip on the above railroad. The invitation was accepted.

A number of recommendations as to the membership of the Association were presented by Dr. Toner, from the Judicial Council, and appropriately referred.

The next business in order was the consideration of the amendment to the Code of Ethics, which was continued from yesterday.

Dr. N. S. Davis, of Chicago, being entitled to the floor, proceeded to reply to the remarks of Dr. Dunster, in an able and lengthy argument, which was greeted with loud laughter and applause.

At the conclusion of Dr. Davis' remarks, the applause was loud and long-continued. The discussion was further continued by Doctors Martin, of Massachusetts, and Dunster, of Michigan.

Dr. Marcy moved to lay the amendment on the table indefinitely.

On motion, the ayes and noes were called, with the following result: Ayes, 106; noes, 108.

The further consideration of the amendment was postponed until to-morrow.

The next business was the consideration of the following amendment to the By-Laws, offered by Dr. J. M. Keller, of Arkansas:

"In the election of officers and appointment of committees by this Association and its President, they shall be confined to members and delegates present at the meeting, except in the Committee of Arrangements, Climatology and Credentials."

The amendment was adopted.

